# Comparative Study of Transcranial Magneto-Acoustic Stimulation and Transcranial Ultrasound Stimulation of Motor Cortex

**DOI:** 10.3389/fnbeh.2019.00241

**Published:** 2019-10-11

**Authors:** Huiqin Wang, Xiaoqing Zhou, Dong Cui, Ruixu Liu, Ruxin Tan, Xin Wang, Zhipeng Liu, Tao Yin

**Affiliations:** Peking Union Medical College, Institute of Biomedical Engineering, Chinese Academy of Medical Sciences, Tianjin, China

**Keywords:** non-invasive neuromodulation, low-intensity focused ultrasound, motor cortex, myoelectric amplitude, myoelectric response latency

## Abstract

Transcranial ultrasound stimulation (TUS; *f* < 1 MHz) is a promising approach to non-invasive brain stimulation. Transcranial magneto-acoustic stimulation (TMAS) is a technique of neuromodulation for regulating neuroelectric-activity utilizing a magnetic–acoustic coupling electric field generated by low-intensity ultrasound and magnetic fields. However, both techniques use the physical means of low-intensity ultrasound and can induce the response of the motor cortex. Therefore, it is necessary to distinguish the difference between the two techniques in the regulation of neural activity. This study is the first to quantify the amplitude and response latency of motor cortical electromyography (EMG) in mice induced by TMAS and TUS. The amplitude of EMG (2.73 ± 0.32 mV) induced by TMAS was significantly greater than that induced by TUS (2.22 ± 0.33 mV), and the EMG response latency induced by TMAS (101.25 ± 88.4 ms) was significantly lower than that induced by TUS (181.25 ± 158.4 ms). This shows that TMAS can shorten the response time of nerve activity and enhance the neuromodulation effect of TUS on the motor cortex. This provides a theoretical basis for revealing the physiological mechanisms of TMAS and the treatment of neuropsychiatric diseases using it.

## Introduction

Various physical stimuli play an increasingly important role in neuroscience. Electricity- (Paulus, 2011; Ziomber et al., 2018), optics- (Perusini et al., 2017), magnetism- (Blumberger et al., 2018), and sound-mediated (Gallay et al., 2016) approaches have emerged as the four main categories of methods used for neuro-stimulation. These techniques of neuromodulation can be divided into two broad categories: non-invasive and invasive. Techniques of non-invasive stimulation, such as transcranial direct-current stimulation (tDCS; Callai et al., 2019) and transcranial magnetic stimulation (TMS; Rotenberg et al., 2014), are characterized by safety and simple operation. However, the spatial resolution of both tDCS and TMS is noticeably worse than that of invasive methods (Zhang et al., 2013; Sánchez-Kuhn et al., 2017). Methods of invasive stimulation, such as deep brain stimulation (DBS; Lei et al., 2015) and optogenetics (Dugué et al., 2011) have characteristics of high spatial resolution and specificity, but they need to be electrode- or fiber-implanted organisms, which increases the operational risk in research and clinical applications (Chen and Zhang, 2009; LaLumiere, 2011).

Transcranial ultrasound stimulation (TUS) is a technique for the neuromodulation of nerve tissue using low-intensity ultrasound. It can achieve high penetration depth and precise focus for neuromodulation of nerves, including deep regions of the brain (Tyler et al., 2018). The ultrasonic frequency of TUS generally does not exceed 1 MHz (Tufail et al., 2011), and its range of intensity is 30–500 mW/cm^2^ (Tyler et al., 2008). The depth of penetration can be varied from the scalp to the entire region of the brain, and the spatial resolution can reach 1~2 mm. Previous studies have reported that TUS can activate the motor cortex (King et al., 2013), visual cortex (Lee et al., 2016), and somatosensory cortex (Lee et al., 2015), and has unique advantages in neuromodulation. What’s more, the depth of focus and penetration of TUS can satisfy the requirements of precise neuromodulation. The thermal effect of low-intensity ultrasound is weak, does not cause thermal damage to normal tissues, and thus is safe for nerve tissue (Kim et al., 2014).

Transcranial magneto-acoustical stimulation (TMAS) is a neuromodulation technique based on the principle of magneto-acoustic coupling (Yuan et al., 2016a). It utilizes the focus and deep penetration of ultrasound to achieve millimeter-scale, spatially resolved, and focused electrical stimulation that directly regulates neuroelectric-activity. The idea of TMAS was first proposed by Norton (Norton, 2003) in 2003, where he noted that it can use the high focus of ultrasound to implement non-invasive electrical stimulation at a high spatial resolution. Simultaneously, He deduced the theory in detail. In 2006, Yang (Yang, 2006) analyzed electrical signals generated by ultrasonic waves as well as their echoes in a static magnetic field and proposed magneto-acoustic coupling can be used for non-invasive detection of nerve current. Li et al. (2015) simulated and experimented with the frequency, amplitude, and distribution of the electric field generated by copper wire samples placed at different positions in the sound field. The induced electric field was shown to be orthogonal to the direction of the sound field and static magnetic field in the sample to be stimulated and was consistent with the distribution of the sound field of the ultrasonic transducer. Subsequently, Yuan et al. (2016b, 2017) and Zhang et al. (2018) used the different neural models to simulate the effect of TMAS on nerves and verify by *in vivo* experiments on animals, and verified that different magnetic and acoustic parameters have an effect on nerve stimulation. In addition, experiments on animals have shown that TMAS can improve the behavior and cognitive ability Parkinson’s disease (PD) mice (Liu et al., 2018; Zhou et al., 2018; Wang H. et al., 2019).

The mechanisms of action of TUS and TMAS are different. TUS uses the mechanical effects of ultrasound to regulate neural activity while TMAS generates a stimulating electric field that directly regulates neuroelectric-activity. However, both utilize the deep penetration and high focus of low-intensity focused ultrasound to realize the precise stimulation of the nerve tissue. TMAS adds a static magnetic field based on TUS so that electrical stimulation is generated by magneto-acoustic coupling while superimposing the effect of ultrasonic stimulation. Previous studies have shown that both TUS and TMAS can stimulate the motor cortex (Wang Y. et al., 2019) and the deep hippocampus (Liu et al., 2018), but no quantitative detailed analysis has been undertaken to date on the effects of the two stimulation methods. This study is the first to quantitatively analyze the amplitude and latency of electromyography (EMG) signals induced by TMAS and TUS, compare the effects of TMAS and TUS on the motor cortex of mice, and to propose the mechanism of action based on the results of current research. This study is important for exploring the mechanism of action and the scope of application of the two stimulation methods.

## Materials and Methods

### Animals

C57BL/6 mice used in this study were purchased from HuaFukang Biotechnology Company (Beijing, China). All mice were housed in a SPF condition with a 12:12 h light-dark cycle and received water and food *ad libitum*. The mice were kept in the plastic cage and the size of which is 32 cm in length by 21 cm in width by 16 cm in height. Each cage contains 3–4 mice. The temperature was maintained at 21~23°C and the humidity was 50%–70%. All experiments on the animals were conducted according to the protocol approved by the Chinese Academy of Medical Science and Peking Union Medical College and were in accordance with the National Institute of Health Guide for the Care and Use of Laboratory Animals (NIH Publications No. 80–23). All measures were taken to minimize the use of animals and the pain they suffered during the experiments according to the request by Biosafety and Animal Ethics. A total of 16 male C57BL/6J (21 ± 2.50 g, 7–8 weeks old) mice were randomly divided into two groups for TUS and TMAS by random number table. The mice were first made to breathe the anesthetic gas (Isoflurane, RWD). Subsequently, fur over their heads and limbs was cropped by scissors and removed by depilation. The mice were then placed in a locator plate with a heating pad to maintain body temperature during the experiments. The head of each mouse was gently fixed on a self-made respiratory fixation device. To ensure the stability of the mouse during the experiment, the anesthetic gas was continuously administered using a breathing anesthesia machine. Ophthalmic ointment was used to protect eyes of the mice from getting dry. Acoustic gel was applied and gently kneaded on the scalp to better couple with the ultrasound probe.

### TUS-TMAS Experimental System

**Figure 1:**
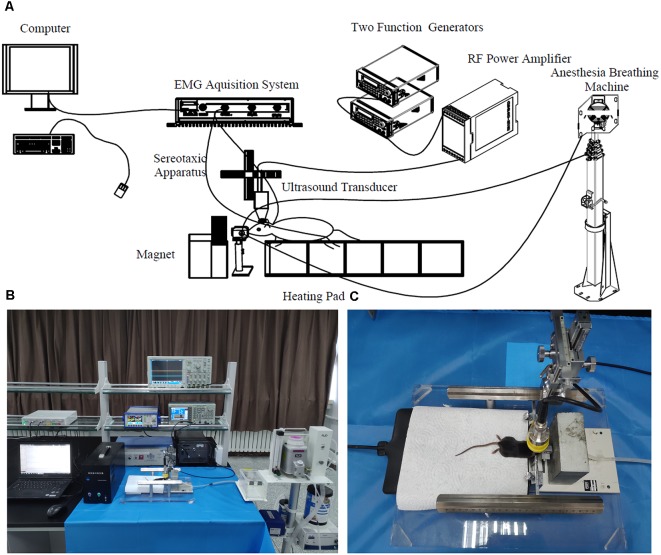
Transcranial ultrasound stimulation (TUS)-transcranial magneto-acoustic stimulation (TMAS) experimental system. **(A)** Diagram of TMAS-TUS experimental system. The ultrasonic stimulation signal is generated by two function signal generators and the ultrasonic transducer is driven by a power amplifier amplified 100 times. The ultrasonic transducer is fixed by the three-axis bracket of the stereotactic locator and the target is stimulated by adjusting the three-axis coordinates. The mouth of the mouse was fixed on a holder with anesthetic gas and its body was fixed on a plate with a heating pad. The depth of anesthesia was maintained by a breathing anesthesia machine. electromyography (EMG) signals of the mouse were collected by a needle electrode and acquired by a multi-channel physiological record acquisition system. **(B)** Map of TMAS experimental platform. The TUS stimulation was performed by removing the magnet in front of the mouse’s head. **(C)** Mouse subjected to TMAS.

To compare TUS and TMAS on *in vivo* mice, this study established a TUS-TMAS integrated experimental system that contained an ultrasound excitation device, static magnet, mouse fixation device, and EMG acquisition and analytical device. The system’s device diagram is shown in Figure 1.

In the TUS-TMAS system, a three-channel signal generator (TFG6920A, Digital, China) was used to generate a pulse repetition signal (PRF), the pulse trigger signal. A four-channel signal generator (AFG3252, Tektronix, Beaverton, OR, USA) was used to generate bursts of specific amplitude and width. The modulated ultrasonic signal was amplified by a power amplifier (HSA4101, NF, Japan) to excite the ultrasonic transducer, and the output of the ultrasonic wave of the transducer was focused by an acoustic collimator to stimulate the target region of the brain of the mouse. The mouse was fixed on a brain stereotaxic apparatus (SR-6M, Chengmao, Japan) and administered mild gas anesthesia in the experiment using a breathing anesthesia machine (R580S, RWD, China). A steady magnetic field was generated by a static magnet placed on the front-loading bracket of the mouse to provide the magnetic field strength required for the TMAS experiment. A multi-channel physiological electrical signal acquisition and processing system (RM6240E/EC, Chengyi, China) were used to collect, amplify, and simply filter EMG signals of the mouse. The experiments on TMAS and TUS in mice were performed by controlling the application and removal of the magnetic field, respectively.

### Ultrasonic Parameter and Magnetic Field Settings

**Figure 2:**
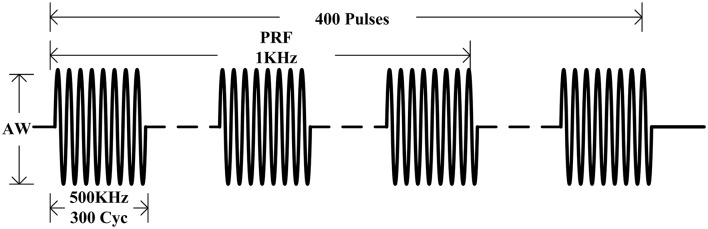
Sequence of ultrasound excitation. The fundamental frequency of the ultrasonic wave (f) was 500 kHz, the fundamental period (Nc) was 300 pulses. The repetition frequency (PRF) was 1 kHz, pulse group repetition number (NTB) was 400, and the period (T) was 4 s.

By referring to the relevant reference which could induce EMG signals (Tufail et al., 2010, 2011; Kubanek, 2018), and considering the safety and effectiveness of ultrasonic intensity, the ultrasonic parameters used in this experiment were as follows: *f* = 500 kHz, TBD = 0.6 ms, PRF = 1 kHz, NTB = 400, and period *T* = 4 s, as shown in Figure 2. The generated ultrasonic signal was amplified 100 times by a power amplifier. The ultrasonic transducer (V301, Olympus, Japan, planar ultrasonic transducer, center frequency 500 kHz) was collimated by a sound collimator (self-made, focal length, 2 mm) and fixed on a stereotactic frame and, by moving along three axes (accuracy 0.01 mm), located the target stimulation area of the mouse. The surface magnetic intensity of the static magnet was obtained by a Gauss meter (Model 475, Lakeshore, USA). The surface magnetic induction intensity was 0.3 T and the magnetic field strength at the target was 0.15 T.

### Selection of Stimulation Target and Sound Pressure Detection

**Figure 3:**
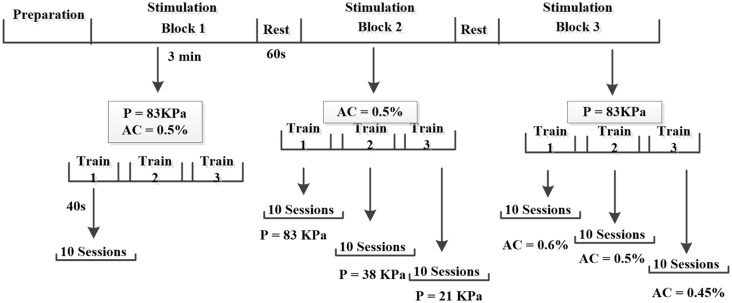
Illustrate of experimental procedures. In the experiment, each mouse was stimulated by three blocks (constant Psptp and anesthetic concentration, different Psptp, different anesthetic concentration) and each block included three trains with 1 min interval. Each train lasted 40 s and 10 EMGs were collected. Furthermore, the preparation included the animal preparation, Psptp detection and pre-test. “P” means Psptp, “AC” means anesthetic concentration.

In the experiment, the target of stimulation was the motor cortex of the mouse, 3.5 mm to the left of the midline of the ear and 7.5 mm behind it (Li et al., 2016). To determine the acoustic parameters of the experiment, a sound field detection experiment at the target was performed before the stimulation. First, the sound field distribution of the ultrasonic transducer was tested to ensure the mice were stimulated at the strongest sound pressure distribution. After that, the sound pressure at the stimulation target was detected. The head of the mouse was fixed on brain stereotaxic apparatus, and the ultrasonic transducer with an acoustic collimator came into contact with the head through the coupling agent to form a stimulation point in the area of motion and recorded the spatial coordinates of the stimulation target. The mice were then removed from the apparatus while the position of the ultrasonic transducer was kept constant. A needle hydrophone (developed by the Institute of Acoustics, Chinese Academy of Sciences, sensitivity 2 μV/Pa) was placed at the stimulation target (head of the mouse) before removal. To ensure test sensitivity, the direction of the needle hydrophone was adjusted so that it was in the same direction as the focused beam of the ultrasonic transducer. An oscilloscope (MSO4104, Tektronix, Beaverton, OR, USA) synchronously detected the ultrasonic transmission signal and the pin hydrophone reception signal, as shown in Figure 3. According to the formula for the spatial-peak temporal-peak acoustic pressure (Psptp)—Psptp = U_L_/M_L_—the true sound pressure at the cortex was obtained under different excitation voltages. U_L_ is the maximum instantaneous value of the measured output voltage and M_L_ is the sensitivity of the hydrophone. According to the Hall effect of the nerve tissue (Norton, 2003), the coupling voltage generated by TMAS can be calculated (the speed of sound in the human brain is set to 1,450 m/s, and the density of brain tissue is 10^3^ kg/m^3^).

### Acquisition and Processing of EMG Signals

The EMG signals were obtained using a pin-type electrode of the multi-channel recording acquisition system. The signals were obtained from the forelimb of the mouse. The positive electrode was inserted into the right forelimb of mouse while the grounding and reference electrodes were connected to the hind limbs, respectively. The pin electrode was connected to the input of the multi-channel recorder, the output of which was connected to a computer. The EMG signals were displayed in real-time on the computer after being amplified and filtered. The main parameters were set as follows: bioelectric mode, 4 kHz sampling frequency, and 1,000 Hz low-pass filtering. The acquired signals were processed in MATLAB. They were first filtered with a 50 Hz notch filter, subsequently, the wavelet transform is used to analyze the EMG signal with db5 wavelet. Finally, the datasets were rectified and sectioned according to the synchronous signal of the stimulus. The peak EMG amplitude was defined as the maximum value in each final dataset. The time between the onset of the stimulus and the active reaction was defined as the response latency (Li et al., 2016). Five EMG signals were selected for analysis once the stimulation had induced a stable EMG signal. The mean amplitude of the five EMGs was defined as the amplitude for each mouse, and the average response latency of the five EMGs was defined as the EMG response latency for each mouse.

### TUS and TMAS Experiments

The TUS of the motor cortex of the mouse was carried out in the TUS system. The ultrasound transducer was moved to locate the motor cortex of the mouse using three-axis stereotaxic instrument, and the power of signal excitation was turned on. To start recording after observing the synchronized and stable motion feedback with the stimulus and observing the real-time stable EMG signals on the computer, the experimental procedure of TMAS was consistent with the experimental procedure of TUS. To form the TMAS system, it was necessary to add a static magnetic field to the TUS system and repeat its operational process to record EMG signals induced by TMAS.

### Comparing Effects of Different Spatial-Peak Temporal-Peak Acoustic Pressure (Psptp)

To study the effects of spatial-peak temporal-peak acoustic pressure (Psptp) on the two stimulation methods, we designed TUS and TMAS experiments under different Psptp by changing the input voltage of the excitation voltage. Using the above ultrasonic parameters (*f* = 500 kHz, TBD = 0.6 ms, PRF = 1 kHz, NTB = 400, *T* = 4 s), the ultrasonic amplitudes were set to 3 V, 1 V, and 0.5 V, corresponding to Psptp of 83 KPa, 38 KPa, and 21 KPa, respectively. The mice were first stimulated with transcranial ultrasound for 3V, and the output voltage was then sequentially lowered to 1 V and 0.5 V. The EMG was collected to analyze its amplitude and response latency.

### Comparing Effects of Different Depths of Anesthesia

To compare the effects of anesthetic concentration on the two stimuli, this study designed TUS and TMAS experiments at different anesthetic concentrations. Using the above experimental parameters, the output voltage was maintained at 3 V, and the concentrations of the gas were 0.6%, 0.5%, and 0.45%. The EMG signals of TUS and TMAS were collected at three anesthetic concentrations, and the amplitude and response latency of EMG were analyzed. The experimental protocol was illustrated in Figure 3.

### Data Analysis and Statistics

Student’s *t*-test (two-tailed) was applied for comparing the amplitude and response latency of EMG between two groups. One-way analysis of variance (ANOVA) analysis was used for comparisons among multiple levels for the factor of sound pressure or anesthetic concentration, followed by *post hoc* analysis (SPSS19.0). All values were expressed as mean ± SE. *P* = 0.05 was accepted as the threshold for statistical significance.

## Results

### The Sound Pressure and Electric Field at the Stimulation Target

We tested the sound pressure of the ultrasound transducer and calculated the electric field generated by magneto-acoustic coupling in the target of mouse’s brain.

**Figure 4:**
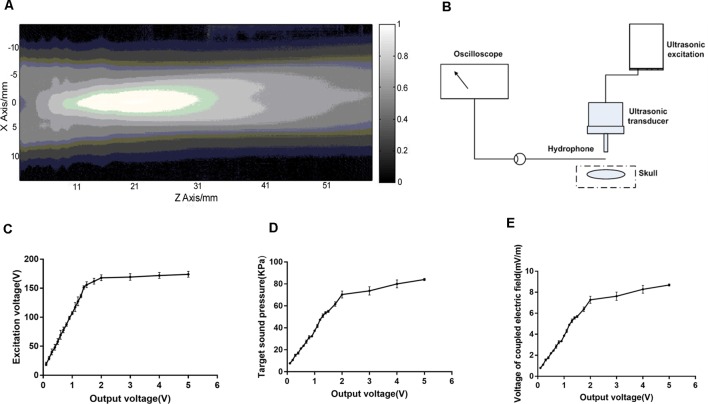
The sound field distribution and sound pressure detection at the target. **(A)** The sound field distribution of the ultrasonic transducer. **(B)** Sound pressure detecting device. **(C,D)** The relationship between the output voltage of the signal generator (500 kHz, 300 pulses, 1 kHz PRF, 400 NTB, *T* = 4 s) and sound pressure of the ultrasound transducer at the target. **(E)** Electric field generated by magneto-acoustic coupling.

The distribution of sound pressure generated by ultrasonic transducer was shown in Figure 4A and the sound pressure detection at the stimulation target was shown in Figure 4B. The region with the strongest sound pressure appeared 1–3 cm in front of the emission plane of the transducer. The stimulus target was at the maximum sound pressure. The relationships between the excitation voltage of the ultrasound transducer and sound pressure at the target were shown in Figures 4C,D. Figure 4C illustrated that the output voltage of the signal generator was linearly related to the excitation voltage of the ultrasonic transducer, but after outputting 2V, the amplifier was saturated and the excitation voltage remained substantially unchanged. The output voltage of the signal generator and sound pressure of the target were linear before saturation (Figure 4D), and the latter changed with the former. Figure 4E showed that the magneto-acoustic coupling electric field is formed by TMAS at the stimulus target.

### Comparison of EMG Amplitude Induced by TUS and TMAS

We first verified whether the TUS and TMAS can induce motor response of mice when the output voltage of the signal generator was 3 V (Psptp of the target was 83 KPa) and anesthetic concentration was 0.5%. In the experiment, EMG signals from the right distal forelimb of mice induced by TUS and TMAS were recorded using a needle electrode with a multi-channel physiological recording system (Figure 1). The mice were continuously anesthetized with isoflurane during the experiment. The ultrasonic transducer was collimated by the acoustic collimator and brought into contact with the mouse’s brain through the ultrasonic coupling agent.

**Figure 5:**
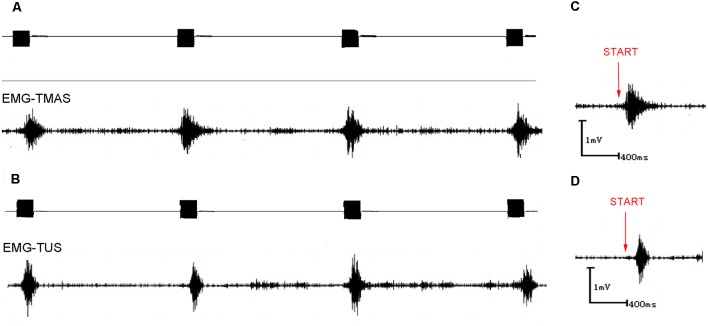
EMG signals induced by TUS and TMAS. **(A)** EMG signal induced by TMAS. Ultrasonic excitation signal (top; 500 kHz, 300 pulses, 1 kHz PRF, 400 NTB, *T* = 4 s); induced synchronous, stable EMG signal (bottom). **(B)** EMG signal induced by TUS. Ultrasonic excitation signal (top; 500 kHz, 300 pulses, 1 kHz PRF, 400 NTB, *T* = 4 s); induced synchronous, stable EMG signal (bottom). **(C)** EMG signal induced by TMAS. Start (red) represents the starting point of the TMAS stimulation. **(D)** EMG signal induced by TUS. Start (red) represents the starting point of the TUS stimulation.

**Figure 6:**
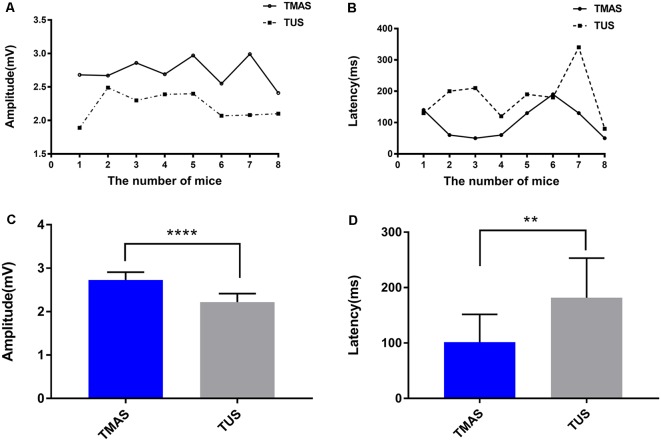
EMG amplitude and response latency induced by TMAS and TUS. **(A)** The two fitted curves are the EMG amplitudes induced by TMAS and TUS at a Psptp of 83 KPa, and each dot represents the average EMG amplitude induced by TMAS in each mouse (*n* = 8). Each square point represents the average value of EMG amplitude induced by TUS in each mouse (*n* = 8). **(B)** Comparison of EMG response latency induced by TMAS and TUS. Each dot represents the average EMG amplitude induced by TMAS in each mouse (*n* = 8). Each square point represents the average value of EMG amplitude induced by TUS in each mouse (*n* = 8). **(C)** Comparison of the average amplitude of TMG induced by TMAS and TUS (*n* = 8). **(D)** Comparison of the EMG average response latency induced by TMAS and TUS (*n* = 8). **p < 0.01, ****p < 0.0001.

Figures 5A,B demonstrated that TMAS and TUS can induce synchronized EMG signals in the mice. Figure 6A showed the comparison of amplitude of EMG induced by the two stimulation methods, and it is evident that the amplitude of the EMG induced by TMAS (2.73 ± 0.32 mV) was higher than that of TUS (2.22 ± 0.33 mV) with the same excitation voltage and anesthetic concentration. The statistical analysis indicated that TMAS and TUS had significant statistical differences (*p* < 0.0001, *independent sample t-test*) in terms of the induced amplitude of EMG (Figure 6C).

### Comparison of EMG Response Latency Induced by TUS and TMAS

Figures 5C,D showed the EMG response latency induced by TMAS and TUS with Psptp of 83 KPa and anesthetic concentration of 0.5%. It is evident that TMAS can induce the EMG signals of mice in a shorter time after the start of the stimulation. The results of statistical analysis (Figures 6B,D) demonstrated that the response latency of EMG induced by the two methods (TMAS: 101.25 ± 88.4 ms, TUS: 181.25 ± 158.4 ms) were significantly different (*p* < 0.01, *independent sample t-test*). This suggested that TMAS can significantly shorten the response latency of EMG and activate the motor cortex of the mice more quickly compared to TUS under the same Psptp and anesthetic concentration.

### Comparison of EMG Amplitudes Induced by TMAS and TUS at Different Psptp

**Figure 7:**
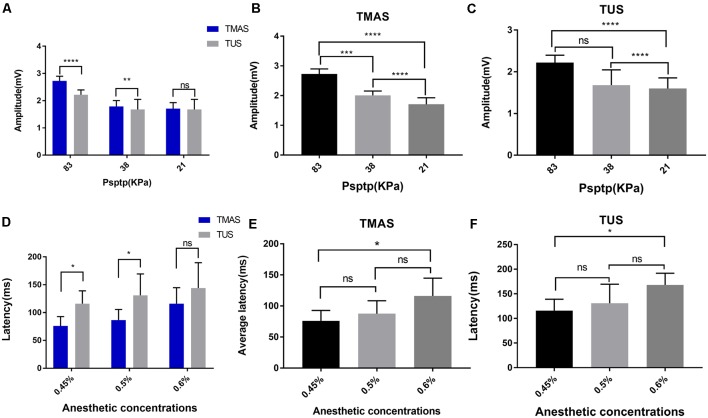
EMG amplitude and response latency of TMAS and TUS at different Psptp and anesthetic concentrations. **(A–C)** EMG amplitude of TMAS and TUS at different Psptp. **(A)** The average amplitude of EMG induced by TMAS (*n* = 8) and TUS (*n* = 8) when the Psptp values were 83 KPa, 38 KPa, and 21 KPa. **(B)** Comparison of TMAS-induced EMG amplitudes when Psptp values were 83 KPa, 38 KPa, and 21 KPa. **(C)** Comparison of TUS-induced EMG amplitudes when Psptp values were 83 KPa, 38 KPa, and 21 KPa. **(D–F)** EMG response latency of TMAS and TUS at different anesthetic concentrations. **(D)** EMG average response latency induced by TMAS (blue histogram, *n* = 8) and TUS (gray histogram, *n* = 8) when the isoflurane gas anesthetic concentrations were 0.45%, 0.5%, and 0.6%. **(E)** Comparison of the average response latency of EMG induced by TMAS at anesthetic concentrations of 0.45%, 0.5%, and 0.6%. **(F)** Comparison of the average response latency of EMG induced by TUS at anesthetic concentrations of 0.45%, 0.5%, and 0.6% [*p < 0.05, ***p < 0.001, one-way analysis of variance (ANOVA), Bonferroni post-test]. **p < 0.01, ****p < 0.0001, ns, means no statistical difference.

To further compare the effects of TMAS and TUS under different values of Psptp, this study designed TMAS and TUS experiments under different Psptp. We collected EMG signals when the Psptp values of the target region for stimulation were 83 KPa, 38 KPa, and 21 KPa at the anesthetic concentration of 0.5%. Figures 7A,B showed the comparison in terms of EMG amplitude induced by TMAS and TUS under different values of Psptp. It is clear that the EMG amplitude induced by TMAS and TUS decreased as sound pressure decreased, but that induced by the former was higher than the latter.

### Comparison of EMG Response Latency Induced by TMAS and TUS at Different Anesthetic Concentrations

This study also verified the effect of different anesthetic depths on the two stimulation methods. We collected EMG signals when the anesthetic concentrations were 0.45%, 0.5%, and 0.6% at a Psptp of 83 KPa (Figure 7D). The results are shown in Figures 7E,F. It is evident that as the concentration of anesthetic gas increased, that is, the depth of anesthesia of the mice increased, the EMG response latency induced by TMAS and TUS increased correspondingly, but that of TMAS was always shorter than that of TUS.

## Discussion

In the present study, we compared the effects of TMAS and TUS on the motor cortex in mice based on a quantitative analysis of the amplitude and latency of EMG signals and demonstrated that TMAS can shorten the response time of nerve activity and enhance the neuromodulation effect of TUS on the motor cortex. The EMG amplitudes induced by TMAS and TUS (Figures 6A,C) show that TMAS can induce stronger EMG signals than TUS, indicating that it can enhance the modulation of TUS on the motor cortex. The results of EMG response latency (Figures 6B,D) have shown that TMAS can significantly shorten the response time of the motor cortex compared to TUS. Previous studies have shown that the responding speed of neural tissue was more quickly to electrical stimulation than acoustic stimulation (Säisänen et al., 2008; Li et al., 2016). Therefore, we hypothesized that the shorter latency of EMG may be due to the magneto-acoustic coupling electric field generated by TMAS stimulated the nerve tissue. Research on TUS has revealed that its neuromodulation may be due to the mechanical effects of sound (Kubanek et al., 2018) because ultrasound can open the cell’s force-controlled Na^+^ and Ca^2+^ ion channels (Tyler et al., 2008), which causes the flow of ions of the cell membrane to produce action potentials, whereas electrical stimulation can directly act on the nerves and its speed of response is faster. In addition, some studies have shown that TUS regulates neural activity through the transmission of auditory pathways (Guo et al., 2018; Sato et al., 2018), which may also be the reason for why the response of ultrasound to the nerve was slower. Furthermore, the results in Figures 6, 7 show that both TMAS and TUS were affected by the intensity of ultrasound and the concentrations of anesthesia in mice. The amplitude and success rate of EMG decreased as the intensity of ultrasound decreased, as well as the response latency of EMG increased as the concentrations of anesthesia increased. Research on TUS has shown that as the depth of anesthesia increases, the success rate of EMG decreases and EMG response latency increases significantly (Yuan et al., 2018), and this study has shown that TMAS followed the same rule. Nevertheless, EMG amplitude induced by TMAS is higher and its EMG response latency is smaller than that induced by TUS under the same experimental conditions. However, when the intensity of ultrasound was too low (Psptp, 21 KPa) or the anesthetic concentration was too high (0.6%), there was no significant difference between the amplitude of EMG and response latency induced by TMAS and TUS, indicating that TMAS can enhance the effect of TUS on neuromodulation a certain intensity of ultrasound and anesthetic concentration. The influence of ultrasound intensity and anesthetic concentration might be significant for the modulation of the motor cortex. TMAS can enhance the effect of TUS on neuromodulation whereas it is not the main factor affecting EMG.

Compared with other non-invasive methods of nerve stimulation, TUS can modulate nerve tissue non-invasively and accurately. However, the biophysical mechanism of ultrasound acting on the nerve tissue remains unclear (Yang et al., 2018), which limits the practical application of TUS to some extent. Furthermore, previous studies have shown TUS can effectively modulate the neural activity of humans and animals, whereas the spatial-peak temporal-average intensity (Ispta) and mechanical index (MI) are relatively high. Even though all of those values are below the US Food and Drug Administration limitations for ultrasound diagnosis safety guidelines (MI = 1.9, Ispta = 720 mW/cm^2^, Isppa = 190 W/cm^2^), it is imperative to identify the safe parameters for TUS applications (Fini and Tyler, 2017) and it is necessary to reduce the ultrasound intensity as low as possible on the basis of the control effect for safety and effectiveness. The magneto-acoustic coupling electric field generated by TMAS can directly regulate the electrical activity of the nerves, and its electrophysiological mechanism acting on the nerve cells is clear. TMAS can realize millimeter-level transcranial electrical stimulation at a high penetration depth by low-intensity focused ultrasound, theoretically. Our study indicated that TMAS could enhance the neuromodulation effect of TUS under conditions of low ultrasound intensity (MI = 0.12, Ispta = 288.9 mW/cm^2^), which meant that TMAS could modulate neural activity more effective and safe. In addition, studies have also demonstrated that both TMAS and TUS can improve the memory and behavioral abilities of PD mice, yet TMAS exhibits superior effects (Liu et al., 2018; Zhou et al., 2018; Wang Y. et al., 2019). These results indicate that the effect of enhancing TMAS on neuromodulation is evident not only in the motor cortex, but also in deep regions of the brain. As a non-invasive and precise method of neuromodulation, TMAS has promising prospects for application.

TMAS is a technology which based on the coupling of low-intensity ultrasound and magnetic fields, therefore, its modulation of neural activity is actually a composite superposition effect by electric field and ultrasound actually. The researches of animals stimulated by TMAS have also shown that TMAS is composite field stimulation, where the synchronized sound field and electric field form a coordinated modulation. The results of this study indicate that TMAS can enhance the modulation of TUS on the nerves, which provides a reference for distinguishing the effects of the two stimulation methods and exploring their mechanisms of action.

## Data Availability Statement

All datasets generated for this study are included in the manuscript.

## Ethics Statement

The animal study was reviewed and approved by Chinese Academy of Medical Science and Peking Union Medical College.

## Author Contributions

HW and XZ prepared the experiment and drafted the manuscript. HW carried out the experiment. XZ updated the experimental setup. DC performed the neurophysiological data analysis and statistics. RL designed the initial experimental system. RT helped carry out the experiment and oversaw EMG data collection. ZL and TY conceived the study and designed the experiments. All authors revised the final version of this manuscript.

## Conflict of Interest

The authors declare that the research was conducted in the absence of any commercial or financial relationships that could be construed as a potential conflict of interest.
